# Early Exposure of Medical Students to a Formal Research Program Promotes Successful Scholarship in a Multi-Campus Medical School

**DOI:** 10.1007/s40670-024-02098-6

**Published:** 2024-06-17

**Authors:** Gloria M. Conover, Mikayla B. Monk, Selina Nigli, Avery Awalt

**Affiliations:** 1 Department of Medical Education, Texas A&M University School of Medicine, Bryan, TX USA; 2 Office of Medical Student Research Education, Medical Research Education Bldg, Texas A&M University School of Medicine, 8447 Riverside Pkwy, Bryan, TX 77807 USA

**Keywords:** Research and scholarship, Self-directed learning, Adaptive expertise, Lifelong learning skills, Communication skills, Community-based medical school, Undergraduate medical education, Research conferences

## Abstract

**Objectives:**

Many physicians today struggle to learn the complexities of the biological basis for evidence-based medicine. To bridge this gap, the Medical Scholar Research Pathway Program (MSRPP) founded in 2019 prepares medical students for analytical reasoning and critical thinking while engaging in faculty-mentored research projects in a community-based public medical school.

**Methods:**

MSRPP is an application-based extracurricular research program, designed for novice and experienced medical students. Three distinct pathways offer ample opportunities for pre-clinical and clinical students to participate in research on a flexible schedule. The program director guides students in one-on-one coaching meetings to set achievable goals with their faculty mentor, and plan their research deliverables, considering their interests and residency plans.

**Results:**

We report the implementation of a multi-year and multi-campus research training program for medical students across five campuses. Our results show that five class cohorts (2021–2025) of MSRPP students were twice as likely to seek formal research support than students not in the program. MSRPP students continuously growth their self-confidence to disseminate their research by practicing oral communication in monthly *Launch talk* research reports and bi-annual research conferences. Moreover, students report they learn technical communication skills and feel inspired to participate in research by interacting with invited faculty seminars. MSRPP students have a significantly higher scholarship output as compared to non-MSRPP students. Early indicators show that students that participate in formal research programs have a measurable difference for matching into competitive residencies.

**Conclusions:**

MSRPP students report that they have gained research competencies and technical skills to establish a baseline to promote their future research engagement. This study provides other peer medical schools with strategies to create the infrastructure to support and promote medical student research.

**Supplementary Information:**

The online version contains supplementary material available at 10.1007/s40670-024-02098-6.

## Introduction

Scholarship and research instruction is a formidable challenge in medical education. Research training programs in diverse learning environments, using elective and required models, with wide variations in teaching methods, scope, and structure, aim to prepare trainees to acquire lifelong learning skills for them to ultimately become better doctors [1]. Under the excellent mentorship of our pre-clerkship faculty at our institution, MS1 learn to critically assess the latest basic translational science literature and, over the course of a semester, write an abstract on a disease topic they have selected [2]. Pre-clerkship students become comfortable with analytical critical thinking that allow them to evaluate the quality and clinical relevance of peer-reviewed medical science journal research articles [3]. Students learn to deliver an in-depth oral presentation that explains the basic science mechanisms underlying a disease pathology [4–7].

Many medical schools across the USA have developed scholarly concentration programs for medical students to facilitate the curricular integration of basic science knowledge with clinical decision-making [7–10]. A national survey revealed that MS1 participating in scholarly concentration programs have a high-baseline interest in research, women placed higher importance on mentorship relationships compared to men, while any student interested in career-long research involvement placed more importance on gaining more research skills and accomplishment-directed goals [11]. A thematic analysis reported that pre-clerkship students perceive the biomedical content taught as relevant and important milestones for their adaptive expertise and professional identity formation [12].

Despite widespread recognition of the value of integrating medical science with clinical practice [11, 13–15], many challenges remain at both the undergraduate and the graduate levels of medical education [16–18]. Mandatory integration of foundational courses with meaningful clinical experiences enriched the learners’ clinical experience using a variety of instructional modalities [19]. Most prominently, in community-based medical schools, typical barriers include lack of protected time during the clerkship curriculum, limited number of trained research mentors, and lack of integration with long-term career planning. Although funding agencies have created bridge and pipeline programs to support future physicians, there are systemic barriers [20]. To begin to address these needs, we report here the design and implementation of the Medical Scholar Research Pathway Program (MSRPP). A formal, yet flexible, voluntary research training program adapted to serve a multi-campus learning community-based medical school environment.

With the advent of artificial intelligence and the access to biomedical information in digital sources, there is an urgent need for medical educators to teach our medical students how to use and appraise biomedicine research information in clinical practice. Indeed, medical education has been moving for decades in the direction of preparing our students to interpret and apply new knowledge to understand the biological processes in patients with unfamiliar symptoms and numerous comorbidities [3]. Another aspect that influences research performance is training medical science educators in the research paradigm in order to improve the rigor of a research output [21].

Herein, the authors describe core program elements of MSRPP, a novel research training spanning 4 years of the UME curriculum. Details about its conception and development process, organizational structure, and mentor and student eligibility criteria are described. Early indicators suggest that our program effectively fosters student research engagement in faculty-mentored experiences, and increased dissemination of scholarship. Students participating in the program readily present research poster and oral presentations and write higher number of peer-review journal articles. Early indicators tracking the impact of our program appear to be associated with positive advantage in matching into highly and medium competitive specialties. Established institutions using mandatory research programs models also find that longitudinal research experiences during medical school increases trainee interest in scholarship and motivates them to pursue a life-long research career [11, 22].

## Materials and Methods

### Survey Data Collection and Statistics

Surveys and logs were collected electronically using Qualtrics software. Raw data were processed using *Excel* spreadsheets prior to plotting charts. The charts and statistical tests were calculated using GraphPad 9.0 analysis tools and verified in the art of science of learning web tool. Consent forms were listed on the cover pages for surveys under TAMU IRB institutional approval for medical education research proposal TAMU IRB 2021-0834 M.

## Interview Logs

Before medical students apply to the program, those interested in pursuing formal research were given information on research opportunities in various ways (office hours, informational sessions the Office of Medical Student Research Education (OMSRE) newsletters, and monthly luncheons). Interested students fill out entry forms to set goals and provide their *Curriculum Vitae* (CVs) to the program coordinator before setting up one-on-one meetings with the program director. Virtual meetings with the program director assess students’ career goals, residency placement goals, and work with the student to integrate research protected time across the medical school curriculum. Pairing students with research mentors has been used in other settings successfully [23]. The meeting logs aid the director to capture information for follow-up coaching. These logs record [1] student information (class, campus, meeting purpose, contact hours, desired residency program, elective plans), and [2] actionable steps that the director recommends according to the students’ educational goals and prior research experience [24]. These notes are used to hold students accountable for their goals. As students define their professional identity or recognize new interests, the program director provides resources to help students realign their research focus at their next session.

## Research Interest Surveys

Biannual surveys were sent electronically four times (2020–2022) to all medical students to determine the research focus baselines of each campus (Bryan, Temple, Dallas, Houston, and Round Rock) and proactively integrate student professional goals with research opportunities. Student enrollment at the various sites is shown in Table 5. The data revealed the baseline perception towards different types of research at our institution without a mandatory requirement for graduation. Also, we tracked campus-specific student research interests, resource availability, and students’ preferred residency choices.

## Research Outcomes

To evaluate the program’s immediate impact on a MSRPP student’s proficiency and output, research deliverables were compared to those of non-MSRPP students with demonstrated interest. Student interest was determined by tracking students that requested information or participated in OMSRE-sponsored events. Research deliverable outcomes measured (1) research report (Launch talk), (2) presentation at internal research conferences Medical Research Colloquium (MRC) and Senior Research Showcase (SRS), and (3) list of peer-reviewed journal articles. These items were collected through self-reported electronic curriculum vitae (CV), as the Open Researcher and Contributor ID (ORCID). Publications were verified using public databases (PubMed and Google Scholar) and sorted into peer-reviewed and non-peer reviewed articles. An analysis comparing research active students that used the research curriculum with those that did not was done using publicly available match lists. Graduates from 2020 to 2023 were sorted by specialty and location and percentages differences were reported.

## Results

The Medical Scholar Research Pathway Program (MSRPP) was established at the Texas A&M School of Medicine in July 2019, as a formal research program. This flexible training program promotes life-long learning by engaging novice and advanced medical students in meaningful research collaborative learning experience.

### MSRPP Program Overview

MSRPP is a faculty-mentored application-based formal research training program that offers medical students three pathways for scholarly research participation. MSRPP students can participate sequentially in three pathways or mix and match according to their interests and mandatory schedule.

The first MSRPP pathway is the Medical Scholar Explorer (MSE) which was designed to support both novice and advanced pre-clerkship students in longitudinal non-credit research introductory opportunity. The Medical Scholar Researcher (MSR) offers an intermediate-level clerkship research opportunity, using elective block(s) scattered throughout the clerkship curriculum. The Distinguished Medical Scholar Researcher (DMSR), a non-credit research opportunity, is primarily targeted for advanced students to pursue full-time external research internships during a gap year after their third year of medical school.

**Fig. 1:**
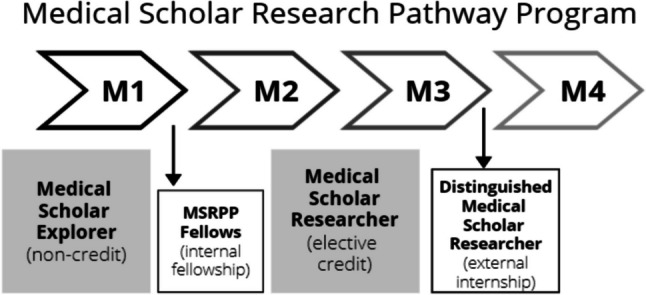
Medical Scholar Research Pathway Program (MSRPP) design. This research training program was tailored for medical students with a flexible longitudinal schedule to accommodate different levels of learners’ aptitudes and interests. Students may select to voluntary engage in three distinct MSRPP pathways faculty-mentored scholarly research during their pre-clinical and/or clinical years. Pre-clinical students typically start with the medical scholar explorer (MSE) pathway, which may or not incorporate the MSRPP Fellows an in-person summer fellowship program that was founded in 2022. Clinical students may decide to participate in medical scholar researcher (MSR) for elective credit and/or formalize an external internship as a year-out of the curriculum as a Distinguished Medical Scholar Researcher Pathway (DMSR)

A major constraint medical students face to participate in research is identifying a suitable mentor and to determine which can fit in their busy mandatory schedule. In 2022, the academic calendar at Texas A&M was changed, opening a 10-week period in the summer. The Office of Medical Student Education (OMSRE) sponsoring MSRPP created competitive research *MSRPP Fellows* internship. MSRPP faculty selected MS2 seeking for a more in-depth full-time research opportunity to engage in-person experiences to pursue clinical or translational research (Fig. 1). Fourteen *MSRPP Fellows* participated in weekly didactic research workshops during full-time 8-week period during the summers 2022 and 2023. In this cohort, we observed students experienced a highly positive and rewarding learning environment for research. Previous literature has shown hands-on summer research training programs increase student self-efficient in research and scholarship [25]. Combined experiential, didactic, and mentoring relationships primed our MSRPP students for continued research practice during clerkship.

### MSRPP Research Pedagogy

**Fig. 2:**
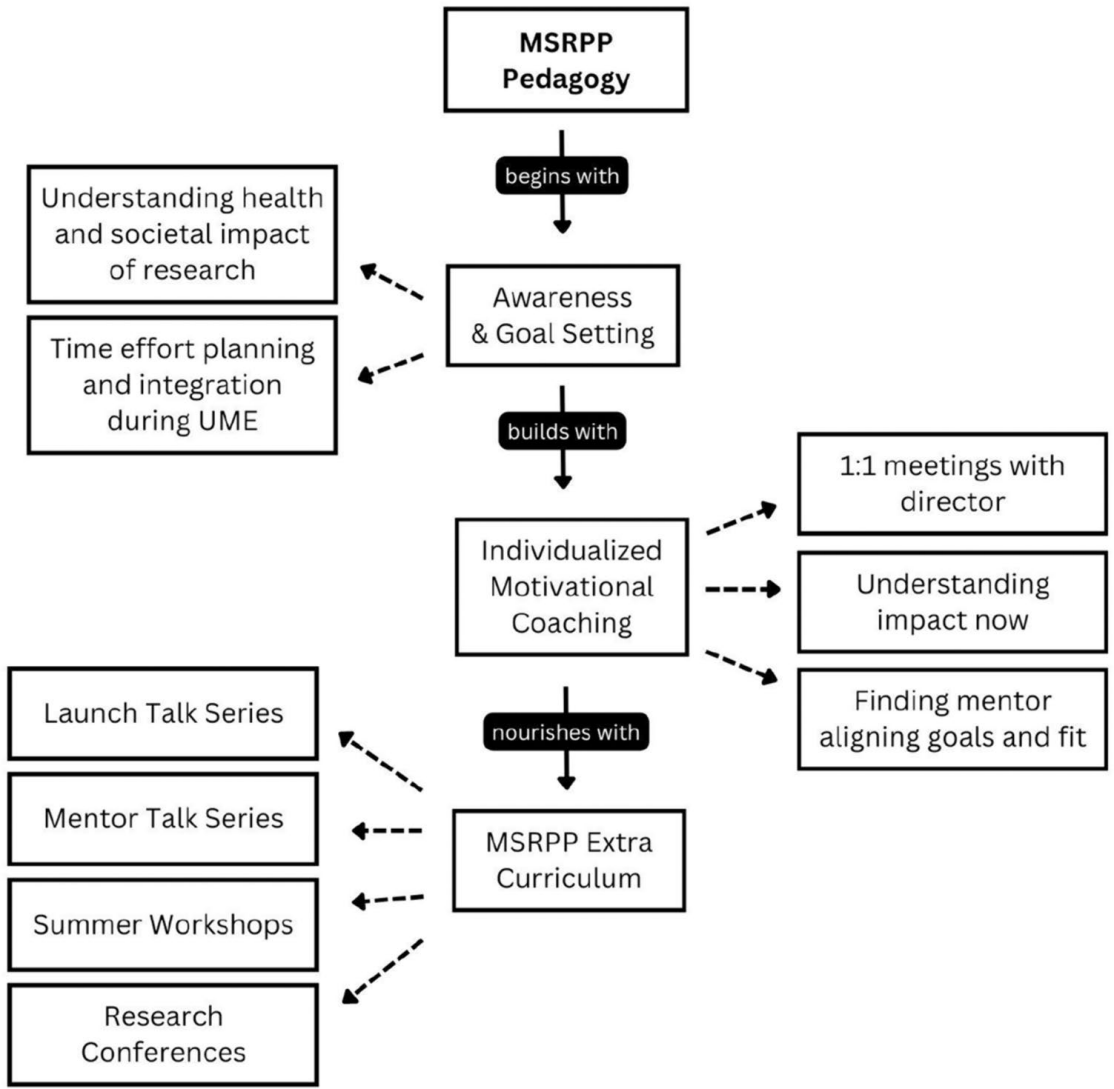
MSRPP instructional concept map framework. MSRPP is a faculty-mentored research non-mandatory training program founded in 2019 at Texas A&M School of Medicine. This framework was custom tailored for our institution based on observation and qualitative analysis of student survey responses (data not shown). This formal program supports research exposure and promotes academic scholarship in medical students attending a community-based medical school with geographically dispersed satellite campuses. Shown is the MSRPP concept map, delineating three phases for research engagement for students to select a pathway that fits and aligns with a student’s career goals. MSRPP students are motivated and coached by the program director to directly reach out to a faculty mentor based on mutual research interests. The MSRPP curriculum entails monthly seminar series given by faculty to inspire and inform students of available research project and capstone research reports “Launch talks” given by MSRPP students. Students seeking to enhance their critical thinking through research exposure can voluntarily decide if they continue or stop at any stage of the process. Research self-directed learning skills and critical reasoning are cultivated in MSRPP students and faculty at bi-annual local research conferences

The MSRPP process begins with student self-awareness and goal setting and builds with multiple individual motivational coaching sessions (Fig. 2). Before applying, students who express interest in scholarly research during medical school request a one-on-one meeting with the program director. Before this meeting, students usually visit with the program coordinator who establishes a rapport with a prospective student. The coordinator may help identify experienced students with prior research experience who may select to pursue the fast-track admission process or novice students who prefer to gather more support following a regular admission process.

Students who seek adaptive learning expertise [26] want a more in-depth coaching experience and proactively take advantage of research curriculum in a regular basis. During the first 2 years, the MSRPP program director had 383 one-on-one research meetings, for a total of 648 contact hours with the students. Specifically, OMSRE recorded 194 meetings in AY2020-2021 and 189 meetings were tallied for AY2021-2022. A tally of the one–one coaching sessions of the program director with students seeking research guidance in this period is shown (Supplemental Fig. 2).

During these meetings, the program director carefully explains to each student the long-life learning career benefits of joining MSRPP using high-quality research advising. After carefully reading the student entry materials, which includes a thorough evaluation of their prior research and experiences, the director listens to student’s self-reported driving motivation to engage in research as a medical student. Subsequently, the program director provides actionable recommendations that are well-aligned with a students’ current research interests, skill sets, and long-term career goals. Finally, the student considers appropriate suggested resources and fit with potential faculty mentors. Of note, students are allowed to pick their own mentor because it increases buy-in and increases success in achieving research deliverables.

Next, the MSRPP student candidate reflects and investigates the research output for potential faculty mentor in a selected discipline. After refining their learning objective for their MSRPP experience, students contact by email a potential faculty mentor to begin a research project by sharing their motivation and requesting an interview. Depending on the need of each student, the director offers individualized advice on the content of the letter the student sends to a potential faculty requesting an interview. From the beginning of the search process, students learn to carefully plan their own experience by deliberately seeking early buy-in from their mentors in the process. Simultaneously, this process prepares medical students for future collaborative real-world research practice and cultivates critical thinking, curiosity, and collaborative teamwork.

Students are admitted to the program when a student, in consultation with their faculty mentor, has concretely delineated a research plan, milestones, and a timeline. For example, to view the MSE application form items, see Supplemental Fig. 1. Because novice students may be unfamiliar with this process, they might need to work on several revisions before their MSRPP application is ready for review. This process teaches students to align their goals with their mentor. Seeking active engagement with the MSRPP mentor, ideally before starting the research project, is an important metric for the success of the research deliverable outcomes. The director verifies that mentors are aware of the educational goals of MSRPP student before they begin the research experience. Students accepted to the program are encouraged to schedule periodic follow-up meetings with the program director to track their progress. One or two meetings per semester with the program director are usually needed to continue research development throughout medical school (see Supplemental Fig. 2). Typically, the MSE pathway lasts the entire pre-clerkship, 18 months before dedicated time for study for their USMLE Step 1, or 2 years after they took the exam to have time to focus on their capstone research report.

### MSRPP Student Enrollment and Geographical Reach

**Fig. 3:**
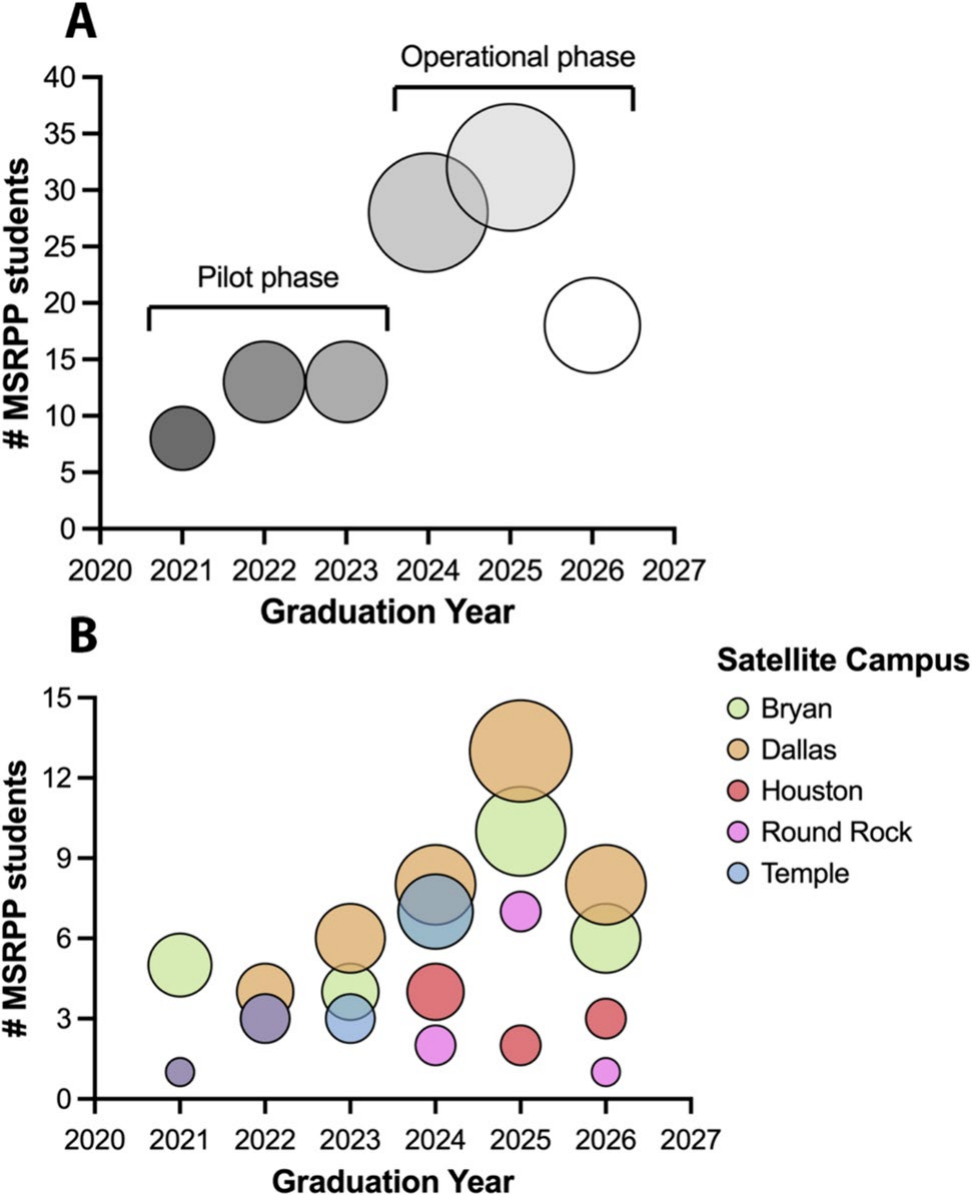
MSRPP student roster and geographical reach during the pilot (AY2019-2020) and operational phase of the research training program (AY 2020–2021, AY 2022–2023). **A** The total number of MSRPP students admitted (*n* = 112) from classes graduating in 2022–2026, 52% were female and 48% were male. A 2.7-fold increase in enrollment was observed as the program transitioned from its pilot phase (classes 2021–2023) to the operational phase (classes 2024–2026). **B** The office of medical student research education (OMSRE) is the primary central research support for Texas A&M medical students. Students have access to affiliate research coordinators when they arrive at clinical campuses. Bubble plots show participation of MSRPP students in five geographically dispersed Texas A&M School of Medicine satellite campuses

MSRPP was piloted with seven MD-only students in July 2019 pursuing single pathways; by December 2023, the program had grown to 112 students pursuing 135 pathways, with 20 students pursuing multiple MSRPP pathways (Tables 1 and 2). This represents an extraordinary ~ 16-fold growth rate, for an extracurricular formal research program, that was launched in the height of the 2020 SARS-CoV2 pandemic. During the first 3 years of the program, 81% of MSRPP pre-clinical students have pursued the MSE pathway (70% regular MSE, and 11% MSRPP Fellows), while 19% clinical students pursued the MSR or DMSR pathways. MSRPP has enrolled a higher percentage of females (52%) than male students (48%) (Fig. 3 and Table 3). MSRPP enrollment tracked from July 2019 to July 2023, spanning the pilot and operational phases, shows that ~ 65% of the students who were advised by the program director decided to move forward and formally *applied* to MSRPP. Of those, 79% started as pre-clerkship students and 21% as clerkship students.

**Table 1:** MSRPP student enrollment. The total number of students in the MSRPP multiple pathways are listed from 2019 to 2023, *n* = 112. Shown are students that completed single or multiple programs (e.g., two pathway programs are denoted double, three pathway programs are denoted triple)

MSRPP program	Single	Double	Triple
Medical Scholar Explorer (MSE)	60	2	0
Team Medical Scholar Explorer (T-MSE)	9	1	0
MSRPP Fellows	10	0	0
Medical Scholar Researcher (MSR)	13	4	1
Distinguished Medical Scholar Researcher (DMSR)	0	3	0
MSRPP Distinction	0	7	2

**Table 2:** MSRPP medical student graduating class distribution. The total number of students in the MSRPP program participating from July 2019 to July 2023 are listed. The table column displays the graduation year of medical students at Texas A&M College of Medicine. Students that completed multiple pathways (*n* = 20) are placed in this table at the highest level of MSRPP program admission. The total number of MSRPP students during this period was *n* = 112

UME level	MSRPP pathway	2027	2026	2025	2024	2023	2022	2021
Pre-clerkship	Medical Scholar Explorer (MSE or Team-MSE)	1	13	19	20	5	10	1
M1/M2 Summer internship	MSRPP Fellows	n/a	7	7	n/a	n/a	n/a	n/a
Clerkship	Medical Scholar Researcher (MSR)	n/a	n/a	1	5	2	3	6
M3/M4	Distinguished Medical Scholar Researcher (DMSR)*	n/a	n/a	n/a	3	NA*	NA*	n/a
Gap 1-year								
3-year program	MSRPP	n/a	4	3	2	n/a	n/a	n/a
	Distinction							

*n/a* not applicable, NA* not available, as the number of DMSR opportunities (gap-year) during the summer 2021 at external institutions was dramatically diminished by the SARS-CoV2 pandemic.

**Table 3:** MSRPP student demographics. The gender distribution of the MSRPP medical student inaugural cohort of MSRPP. The percentages were determined using total number of students in the program (*n* = 112)

Gender	2026	2025	2024	2023	2022	2021
Female	6 (5.4%)	15 (13.4%)	18 (16.1%)	7 (6.3%)	7 (6.3%)	5 (4.8%)
Male	12 (10.7%)	17 (15.2%)	10 (8.9%)	6 (5.4%)	6 (5.4%)	2 (1.9%)

Having multiple, geographically dispersed campuses is both a challenge and an opportunity. Although MS1 met the program director and staff during their first year of medical school in Bryan, after they start their third semester, they become dispersed in five satellite campuses. For the 2022–2025 classes, Dallas was the campus with most MSRPP research interest (35%), followed by Bryan (19%). Other sites Temple (16%) closed for the Class 2025*.* Note that Round Rock campus was open for Class 2022 and 2023 (10%) but closed for Class 2024 reopening for Class 2025 and 2026. Houston (14%) began its independent intercollegiate EnMed track in 2019 in downtown Houston, allowing only students from Class 2021 and 2022 to participate in MSRPP while opening a second clerkship site open in north Houston last year (Tables 1 and 4). Regardless of the satellite campuses status, MSRPP students were reached by OMSRE both by individual videoconference and by travel of the staff to the regional campuses (Fig. 3B, Table 5).

**Table 4:** MSRPP didactic research curriculum. These curricular offerings were created, hosted, and managed for MSRPP students and any matriculated medical student interested in presenting at the research conferences. Four research major educational resources were tracked and hosted by OMSRE under the founding program director leadership from 2020 to 2023. These resources were faculty mentor talks, MSRPP student research reports *Launch talks*, MSRPP Fellows workshops or invited Keynote Clinical Researcher Speakers Medical Research Colloquium (MRC) or Senior Research Showcase (SRS) internal medical research conferences. The percentages were calculated using the total number of people participating per category. A total number of TAMU research medical students (*n* = 106) participated in the research curriculum sponsored and managed by centralized OMSRE dedicated leadership

OMSRE Curriculum	Faculty Talks	Launch Talks	Fellows Workshops	Conference Keynotes Speakers
Clinical Science	8 (7.5%)	26 (24.5%)	7 (6.6%)	6 (5.7%)
Translational Science	13 (12.3%)	15 (14.2%)	2 (1.9%)	0
Research Methods	3 (2.8%)	3 (2.8%)	14 (13.2%)	0
Other (humanities, social sciences)	2 (1.9%)	6 (5.7%)	0	1 (0.9%)

**Table 5:** TAMU medical student geographical distribution. Matriculated students at Texas A&M College of Medicine clerkship campus assignment during years 2018–2021 representing student in graduating classes 2022 through 2025. The total number of students matriculated in this period (*n* = 505). Note that these totals were used to generate the bubble plot sizes in Fig. 2

TAMU Medical Student Matriculation (Graduation) Years	Bryan	Dallas	Houston	Round Rock	Temple
July 2021 (Class 2025)	34	56	25	50	0
July 2020 (Class 2024)	18	46	26	10	34
July 2019 (Class 2023)	17	31	4	0	30
July 2018 (Class 2022)	19	43	32	0	30

**Fig. 4:**
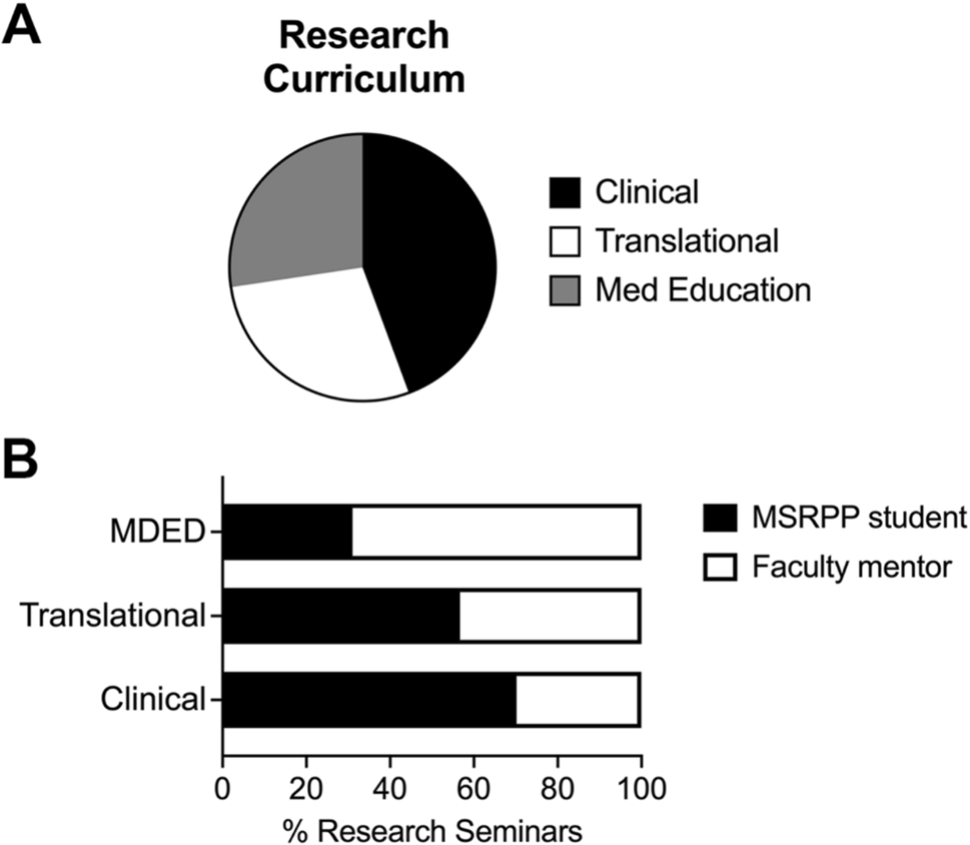
A deliberately designed research curriculum for medical students supports growth and scholarly activity competency attainment. **A** Pie chart displays instruction of three types of research seminars (Clinical, Translational, and Medical Education) open to all medical students and faculty mentors at our institution. Since September 2020, the Office of Medical Student Education (OMSRE) has centrally sponsored and organized monthly MSRPP student-led research reports and research-active faculty seminars. **B** MSRPP students preferentially chose to pursue research and deliver their research reports in clinical topics (55.9%) followed by translational research (28.8%). In contrast, research faculty invited to give instruction to research students delivered didactic seminars to enhance scientific method and scholarly research best methods and practices (42.6%)

During the first 3 years of MSRPP, 92 MSRPP students completed one pathway, 17 completed two, and 3 completed three (Table 1). Two students that previously completed an MSR decided to pursue the DMSR pathway. To date, MSRPP has enrolled 112 students in its various programs and its program director facilitated formal research mentorship with ~ 82 clinical and academic faculty members across our 5 regional campuses (Fig. 3, Tables 1 and 2). Notably, 20 of these students chose to deepen further their critical thinking and reasoning skills applied to their research by pursuing two or more MSRPP pathways (Table 1). Of the ones that completed multiple pathways, six students presented multiple research projects in the launch talk series (Fig. 4 and Table 4).

### MSRPP Curriculum: Research Seminar Series and Local Conferences

As part of this program, MSRPP students are highly encouraged to present their research under faculty mentor guidance as capstone presentations in the *Launch Talk* seminar series (Fig. 4). This venue offers students a unique opportunity to present their progress report and discuss challenges and future directions of their research. Students get comfortable disseminating their scholarly research in a formal environment and receive live feedback from their peers and faculty. To date, students have delivered 68.4% MSE talks; of those, 3.5% were team MSE and 10.5% were MSRPP Fellows. For clinical students, 12.3% MSR talks and 5.3% DMSR students were delivered (Fig. 4, Table 4).

To increase access and foster collaboration, across the five satellite campuses, to date, 58 *Launch Talks* have been held through virtual video conference (Figs. 3 and 4). Topics range from clinical and translational science to graphic medicine and medical education. The majority of students delivered *Launch talks* about clinical research topics followed by translational research (Fig. 4A, Table 4). This is in line, not surprisingly, with our findings of a campus-wide baseline survey that revealed that our medical student body overwhelmingly preferred clinical research projects (Supplemental Fig. 1). In contrast, most of faculty invited delivered didactic research seminars to cultivate in students’ best practices for the scientific method, research processes, and research dissemination methodology (Fig. 4B, Table 4).

Although many students met the *Launch Talk* milestone, any medical student interested in research was invited to participate in OMSRE-hosted research conferences. We offer two annual poster competitions one in the Spring Medical Research Colloquium (MRC), and another one in the Fall Senior Research Showcase (SRS) where students receive live feedback on their research design, methodology, and conclusions in original research and case report categories. Both conferences give opportunities to strengthen MSRPP student and others technical oral communication skills and an opportunity to be inspired by their co-presenting peers.

### MSRPP Research Resources

Students were effectively advised and benefited tremendously from the formative feedback that they received in the 58 MSRPP student virtual *Launch talks* and 49 faculty mentor talks across geographically dispersed urban and rural clinical settings (Figs. 3B, 4B and Table 4). Data show a dramatic increase of interest in MSRPP starting with the Class of 2024 and reaching an increasing interest with the Class of 2025. It is our experience that novice students in research tend to wait until their second year to get involved through in a formal program. Thus, it will take longer to follow-up and determine if the research extracurricular interest continues to grow at our institution.

### Tracking MSRPP Student Research Output

**Fig. 5:**
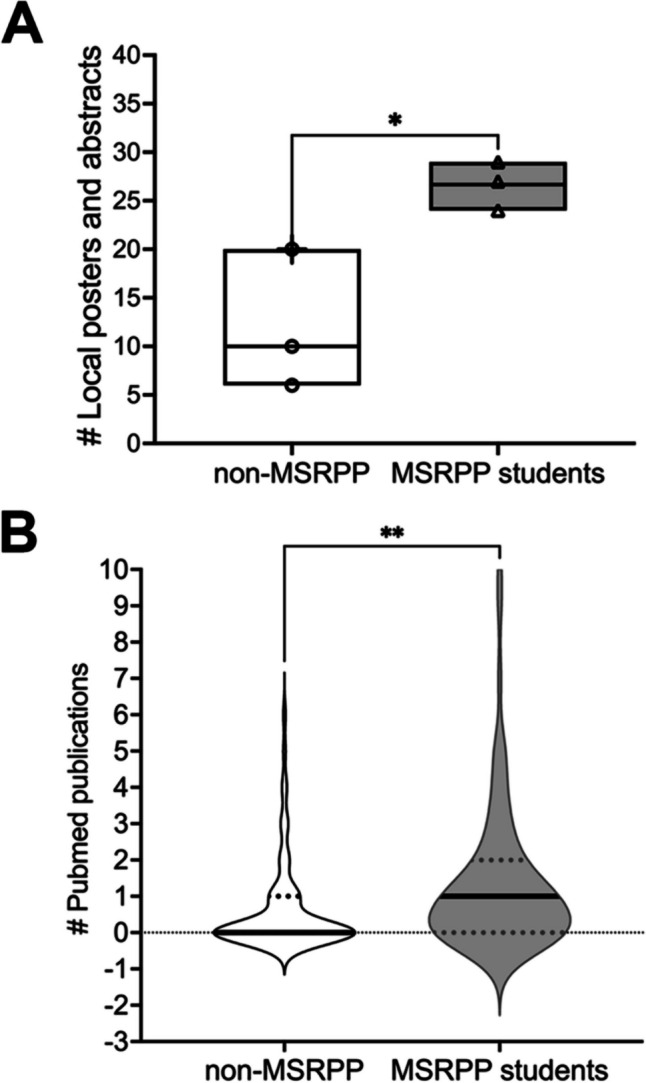
Higher numbers of research deliverables are produced by MSRPP students compared to those who contacted OMSRE but decided not enrolled in the formal research program. **A** MSRPP students (*n* = 80) presented more posters and abstracts than non-MSRPP students (*n* = 36) compared to those who presented but did not choose to formalize their research experience. Poster papers were presented in the Medical Research Colloquium (MRC) or Senior Research Showcase (SRS) competitions hosted by OMSRE. Data was collected for both groups from classes graduating 2023 to 2025 and is displayed in the box plot (unpaired *t*-test, *p-value = 0.0292). The 95% confidence interval is 2.424 to 26.91. **B** More peer-reviewed publications were authored by MSRPP students (*n* = 86 students produced 134 publications) compared to those who chose not to apply to the program (*n* = 80 students produced 11 publications) from classes 2022 to 2025 and is displayed distributions in the violin plot (unpaired, 2 sample *t*-test, **p-value = 0.0054). The 95% confidence interval is 0.214 to 1.206

We began to measure the 3-year research output of the MSRPP students (currently enrolled students and recent Alumni) by tracking their research deliverables by using two metrics. The first measured the number of local poster or abstract presentations, and the second counted the number of peer-reviewed publications (Fig. 5A, B, respectively).

A significant higher number of research deliverables for the MSRPP students was found (up to tenfold) to increase compared to that for the non-MSRPP students. The research output (posters and publications) of MSRPP students was statistically higher compared to that of the other group (non-MSRPP students) who had expressed interest in research (e.g., students defined in latter group by requesting advising meetings and/or participating in local research conferences) but chose not to apply to the program. Each publication for both groups was retrieved from searching in publicly available databases (PubMed and Google Scholar). Publications were also cross-verified if the student had a current ORCID profile. Our results suggest that MSRPP students who had access to high-quality research mentoring achieved higher number of research deliverables compared to students that chose not to apply to the program (e.g., non-MSRPP students).

These results likely occurred because MSRPP students were provided centralized institutional support to find a mentor, and established a mentoring relationship with their mentor after carefully planning the experience. This may explain the higher research output, both in number and in quality, of their research deliverables. Internal conferences provide opportunities to strengthen students’ oral communication skills and inspire all students to present their research progress reports. Furthermore, MSRPP students felt that they belonged to a research community of scholars supported by their research faculty mentors and continuously learned from their peers while attending research seminars (Fig. 2).

Overall, we found that MSRPP students readily take advantage of OMSRE central research-focused resources and are highly motivated to fulfil their research-focused milestones outlined in their MSRPP application (Supplemental Fig. 1). In addition, MSRPP students feel that the positive mentorship relationships they experienced contributed to their research competency development enhancing their perceived competitiveness for residency match. The higher research output for MSRPP students observed here indicates substantial gains in research competencies (e.g., scientific method, analytical reasoning, and communication skills).

### Impact of Research Participation on Residency Placement

**Fig. 6:**
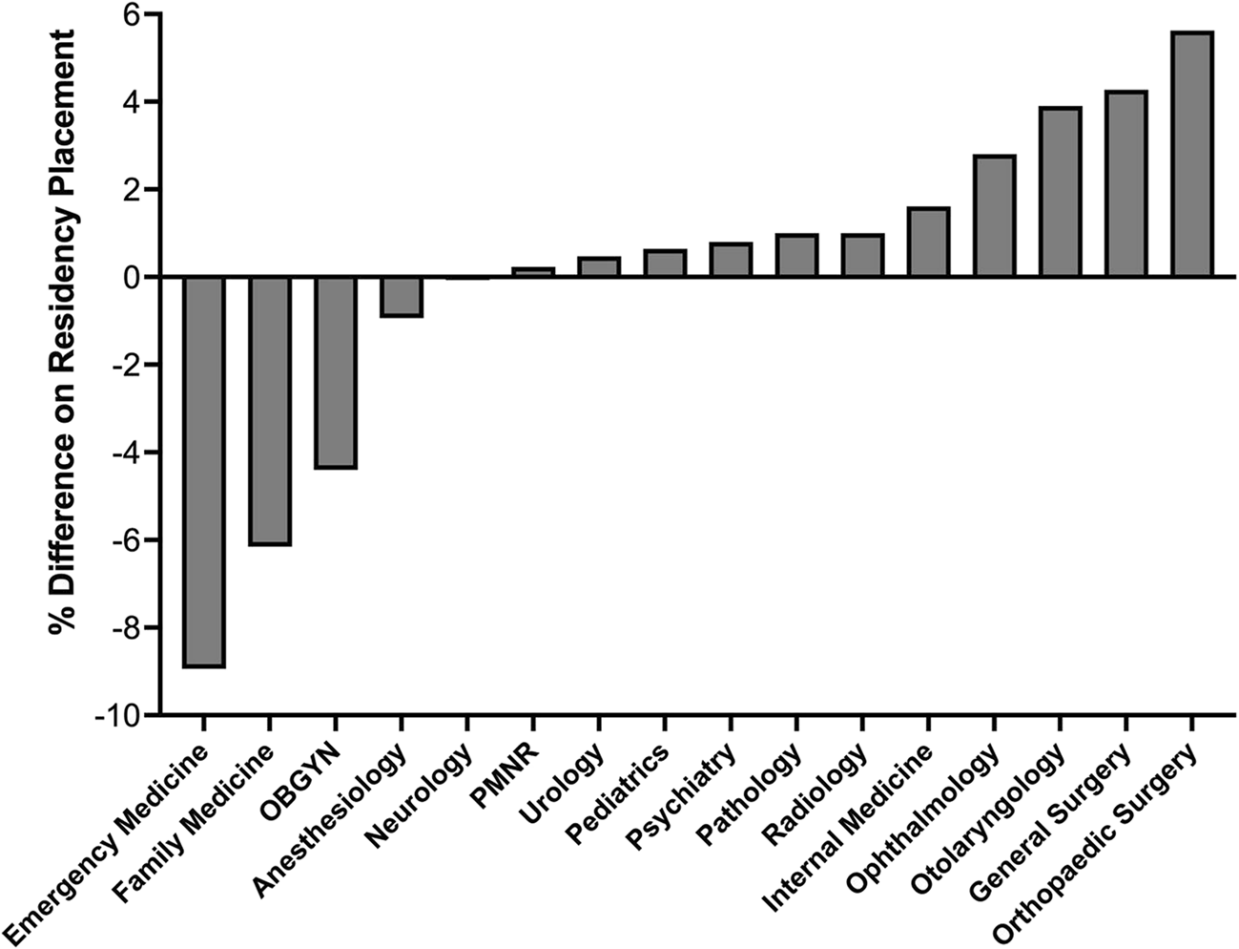
Impact of UME research training on residency placement for TAMU MD graduates. Publicly available match lists of recent MD graduates from 2020 to 2023 were sorted into specialties comparing research active (*n* = 96) and inactive graduates (*n* = 372). This analysis excluded MD-PhD graduates and those who matched to preliminary and transitional year programs. Research active students were identified as those that participated in the Office of Medical Student Research Education (OMSRE)–sponsored research training programs. The bar graph shows the difference in percentages among formal research active (OMSRE users) and research inactive MD graduates, who did not use centralized institutional resources and were defined in our study as inactive. A positive trend of research participation was measured in 68.8% (*n* = 11) specialties, while no effect was apparent in 6.3% (*n* = 1) specialty and a negative one in 25.0% (*n* = 4) specialties. For comparison, historical data shows that graduates from 2018 (prior to the foundation of MSRPP) showed moderately lower percentages of students who matched to general surgery and internal medicine, while no graduates from 2019 matched to ophthalmology

To begin to decipher the impact of UME research training on residency placement at our institution, we tracked residency placement for students utilizing OMSRE services compared to students who did not who graduated from 2020 to 2023 (Fig. 6). Publicly available match lists were sorted by specialty comparing research active (*n* = 96) to inactive graduates (*n* = 372) excluding MD-PhD graduates and those who matched to preliminary and transitional year programs. Our analysis compared the overall percentage difference on residency placement during the study period (MD graduates 2020 to 2023) at the time where the formal research program MSRPP had just been launched at our institution in 2020. This preliminary analysis revealed a small but positive impact on 68.8% of 11 specialties measured (Fig. 6). The most pronounced effect was found for orthopedic surgery (5.6%) and general surgery (4.3%) followed by otolaryngology (3.9%) and ophthalmology (2.8%). During the 2020–2023 period, 47.9% of all MD graduates at Texas A&M matched to national programs while 52.1% matched to Texas (Supplemental Fig. 2).

Within the inaugural cohort of MSRPP alumni, 31 students were matched to 13 distinct medical specialties with 69% of them in the most competitive ones (otolaryngology, ophthalmology, dermatology, pediatrics, obstetrics, and gynecology) or middle competitive ones (emergency medicine, radiology, surgery) as defined by previous studies [27]. This data suggests that research participation in medical school appears to be an important aspect of perceived strength in an applicant, even in graduates before Step 1 transitioned to pass/fail. Moving forward without a numerical grade for Step 1, a recent survey of residency program directors, indicated that applicants with a meaningful research participation demonstrate intellectual curiosity, critical and analytical thinking, and self-directed learning skills [27].

## Discussion

### MSRPP Student Motivational Coaching: Awareness and Goal Setting

Medical students pursuing different MSRPP pathways were effectively advised by a program director to plan a meaningful research experience while they completed medical school. By helping a student define their intent and clarify their present motivation in writing, they learn to strategically identify potential research mentors and interesting projects (Figs. 3 and 5). The program director used a modified FUEL coaching framework to guide students in selecting their faculty mentor as described by Zenger and Stinnett [28]. Coaching meetings occurred as needed by students using video calls. Early participation in research prepares students to carve out time in their schedule for a longitudinally research experience during pre-clerkship. We observed that MSRPP students intentionally advance their careers while developing their research skills throughout their 4 or 5 years in medical school. For example, while completing their research projects, MSRPP students rapidly appreciate that collaborative research teams can more effectively tackle complex problems with numerous comorbidities, making them appreciate interdisciplinary professional interactions early in their medical education.

### Communicating and Modeling Medical Student Research Best Practices

Our MSRPP faculty mentors and students may be in the same location or geographically separated; however, they can access high-quality research content, and receive formative feedback on their research via annual virtual conferences and monthly talk series (Figs. 2 and 3). Establishing a robust foundation for learners to become comfortable and proficient in research and scholarship reasoning skills was also accomplished by inviting eight Keynote physician speakers with active research platforms to lead the bi-annual conferences. Physicians delivered research content with clear impact on patient care which highly motivated novice and advanced research-focused medical students.

MSRPP students present monthly capstone formal talks (*Launch Talks*) and learn by observing best practices from their peers and participating in discussion afterwards. Moreover, students in the program seek out ways to increase their oral poster presentation before attending national professional medical meetings by participating in OMSRE-sponsored student and faculty seminars, and *MSRPP Fellows* summer workshops (Fig. 4 and Table 4). MSRPP students at our institution have access to excellent opportunities to strengthen their desired research focus (clinical, basic or translational science, quality improvement, and medical education). Students are guided primarily by their specialty interest and are motivated to design a research plan that can be conducted asynchronously on a longitudinal schedule.

### MSRPP Increases Student Research Deliverable Output

A key instructional goal for MSRPP students, as discussed above, is to improve their baseline oral communication skills by preparing and giving research reports on their MSRPP research (Fig. 4). A secondary goal is for medical students to produce high-quality peer-reviewed articles with the support of their faculty mentors (Fig. 5). The type of research produced by MSRPP students spans peer-reviewed case reports, original research, and review articles. Prior studies show that local research symposia in community-based settings (where resources are more difficult to access) improve resident and medical student abstract submission and IRB-approved projects [29].

Student participation presenting research at internal research conferences has a particular impact on research-naïve students. Participating in the conference allows them to better appreciate the impact of research on medicine which concrete examples. Throughout their participation in the program, students learn to define and refine research questions and use appropriate methodology while strengthening their oral and written communication skills.

### MSRPP Promotes Research Productivity

MSRPP students published more peer-reviewed articles as compared to those not participating in the program (Fig. 4). The research curriculum engages students in self-directed research productivity, and attributes consistent with developing master adaptive life-long learning [1, 29]. Testimonials gathered reveal high student satisfaction with the program. A student stated, “during my MSRPP MSE research experience, I learned how to independently gather and analyze data, translate findings into written word, and concisely present information.” Similarly, another said “Having this agency and ownership over the research really helped me develop my own mind and gave me a better sense of what it means to really do research.” Medical student research experiences in MSRPP are similar to those reported in mandatory research year programs abroad [30], because students perceive MSRPP as meaningful program that enables them with opportunities to learn new research skills under active-research faculty guidance.

### Research Training Program Limitations

One limitation of this study is that MSRPP was developed and implemented at a single community-based medical school with satellite campuses. Although pre-clerkship MSRPP students are aware of the virtual coaching resources offered through OMSRE, it is challenging to maintain consistent advising for students among five satellite research coordinators. Often affiliated staff report being overwhelmed with the high number of requests from students at their clerkship campuses wanting to get involved in research and the limited number of clinicians investigators available at their sites to mentor research-focused clinical projects. Another limitation is the number of research-active affiliate clinical faculty teaching in community hospitals. Without formally training clinical faculty in scholarly research process, untrained faculty can derail or slow a trainee’s scholarly research development. A third limitation is the lack of long blocks for dedicated time for research between clinical rotations. This challenge can be bypassed by some MSRPP students who decide to apply, prepare, and successfully obtain external competitive fellowships to do 1-year dedicated research program (Supplemental materials, Table 2).

Given that USMLE Step 1 numerical scores are no longer reported correlation with success in matching to residency programs, UME programs may likely increase efforts to offer more centralized support for formal research programs customized for medical student needs [27, 31]. The physician-scientist training programs contribute about 1.5% of the total physician workforce in the USA [17, 32]. Thus, expanding training programs to future physician practicing skilled in collaborative research practice is imperative to meet the US healthcare needs. MSRPP offers medical students and faculty mentors a relatively low-cost formal UME research training program that can be implemented in other medical schools.

### Future Directions

This study will aid other community-based or low-resource medical schools to set curricular priorities to prepare their medical students to be proficient in life-long meaningful research participation. Additionally, students participating in a formal research program are more likely to experience how professional collaboration occurs in real-time scenarios that improve healthcare outcomes. Finally, it is conceivable that in the future, programs such as this could be adapted to implement cost-effective intervention via distance learning to make an educational impact in training global healthcare professionals.

A MSRPP Distinction for advanced students, seeking deeper engagement in research, was piloted in the Fall 2023 with nine students. This 3-year distinction program intentionally integrates foundational sciences into the clinical curriculum to increase instruction of evidence-based clinical reasoning [19]. Hence, long-term follow-up studies could determine MSRPP impact on alumni research productivity, residency placement, and its relationship to success in obtaining grant funding. A decade-long study may also evaluate patient outcomes cared by MSRPP Alumni who actively engaged in clinical translational research in collaborative teams.

## Conclusion

The Medical Scholar Research Pathway Program (MSRPP) was originally designed and implemented for medical students at Texas A&M to enhance their access and meaningful engagement in research and scholarship. This study describes how research educational resources were leveraged and overseen from a central research office reaching students virtually in five satellite clerkship regional campuses. After completion of MSRPP, in their capstone launch talks, student testimonials reveal high confidence to participate academic research. Furthermore, students in the program report they feel they have increased their baseline master adaptive learning research skills and feel ready to establish and sustain productive research collaborations.

## Supplementary Information

Below is the link to the electronic supplementary material.

Supplementary file1 (DOCX 423 KB)

## Data Availability

Raw data were generated at Texas A&M University. Derived data supporting the findings of this study are available from the corresponding author GC on request.
