# Correction to: Early Exposure of Medical Students to a Formal Research Program Promotes Successful Scholarship in a Multi-Campus Medical School

**DOI:** 10.1007/s40670-024-02110-z

**Published:** 2024-07-15

**Authors:** Gloria M. Conover, Mikayla B. Monk, Selina Nigli, Avery Awalt

**Affiliations:** 1 Department of Medical Education, Texas A&M University School of Medicine, Bryan, TX USA; 2 Office of Medical Student Research Education, Medical Research Education Bldg, Texas A&M University School of Medicine, 8447 Riverside Pkwy, Bryan, TX 77807 USA

**Correction to: Medical Science Educator**

10.1007/s40670-024-02098-6

The article “Early Exposure of Medical Students to a Formal Research Program Promotes Successful Scholarship in a Multi-Campus Medical School”, was originally published electronically on the publisher’s internet portal on 17 June 2024 with error in second authors' affiliation. 'Texas A&M College of Medicine' should be removed. The second affiliation should read as follows:

Office of Medical Student Research Education, Medical Research Education Bldg, Texas A&M University School of Medicine, 8447 Riverside Pkwy, Bryan, TX 77807, USA

In addition to this update, supplementary file will be replaced with updated file.

The original article has been corrected.

