# Supplementary material for: Early Exposure of Medical Students to a Formal Research Program Promotes Successful Scholarship in a Multi-Campus Medical School
Source: Med Sci Educ. 2024 Jun 17;34(5):1091–103. doi: 10.1007/s40670-024-02098-6 (PMC11496467; doi:10.1007/s40670-024-02098-6)
Supplement: Supplementary file 1 — Supplementary file1 (DOCX 423 KB) [file 40670_2024_2098_MOESM1_ESM.docx]

**Supplemental Information**

**MSRPP Students Expectations for Research Skill Development**

Since its foundation in 2019, MSRPP was conceived for all medical student with or without prior research experience wanting to pursue formal research during medical school. The program to-date has accepted beginner, intermediate and advanced level medical students that have been successfully matched to 92 faculty mentors. All accepted MSRPP students complete an application that serves as a student-mentor agreement that delineates in writing the learning objectives, expected timetable, expected research presentations and research deliverable plans. In addition to the logistical portions of the application, the student writes an abstract that provides a synopsis of their MSRPP research project providing enough clinical context to explain how the research is expected to move forward an unmet healthcare need. The technical skill developed by each student while in the program is variable, and it depends on the faculty engagement, the student effort, and the research scholarly opportunities they decide to pursue. As an example, for other institutions interested in adapting MSRPP-like program in their schools the medical scholar explorer (MSE) form is included.

**History of Building the MSRPP Support Infrastructure**

For medical schools, interested in launching a formal research program to support medical student research, below we describe a synopsis covering details of the design, pilot, and implementation phases for MSRPP. In the spring of 2019, the founding program director, conceived and designed, a flexible formal research program that allowed medical students to explore research, participate according to their interest, and produce and disseminate scholarship during their four years of their undergraduate medical education.

The first award launched for this new educational initiative, was the Medical Research Travel Award (MRTA). This competitive award was offered twice a year, at the end of each semester, to incentivize MSRPP student research presentation and professional networking at national meetings. Since its foundation in 2019, 142 medical students have successfully completed the application that serves as an agreement between research mentor and student outlining the goal, timeline, research deliverables and plans prior to starting the research experience (see example, Medical Scholar Explorer, Application, Supplemental Figure 1). In 2020, creation of monthly student research reports and mentor talks across a wide breath of medical disciplines gave concrete examples of research program that contribute to development of treatments. That same year, internal research conferences competitions were launched, as a major infrastructure tailored for medical student researchers, to enhance a regional multi-campus network community of scholars. Best poster awards, voted by the faculty judges, boosted students’ confidence and competency to formally present research, preparing them for national meetings. Invited clinical keynote speakers modeled research best practices. A synopsis of MSRPP educational resources is available [here](https://m.youtube.com/watch?v=zKYY4LS6FdY&feature=youtu.be).

| 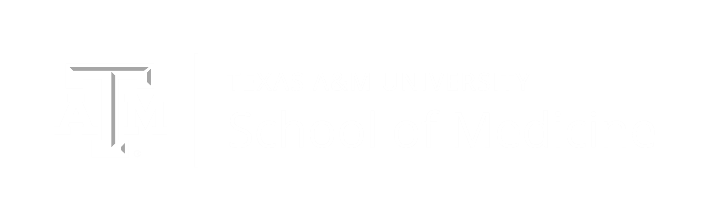 | MSRPP  *Medical Scholar Explorer (MSE)* |
| --- | --- |

Medical Scholar Explorer (MSE) Application

The Medical Scholar Explorer (MSE) pathway is a voluntary *non-credit* scholarly research educational experience available to Texas A&M medical students in good academic standing that give skills and tools for students to advance their long-term professional research development plans.

Once a research mentor has been identified, with the guidance of our Office career along with coaching and mentoring services, medical students will complete the appropriate trainings (TAMU TrainTraq and/or collaborative institutional training initiative). For more detailed application steps visit our [OMSRE website](https://medicine.tamu.edu/omsre/index.html).

Submit your completed MSE form to med-omsre@tamu.edu with a copy to the Director of Medical Student Research Education, Dr. Gloria Conover [gconover@tamu.edu](mailto:gconover@tamu.edu) and our program coordinator, Dr. Selina Nigli, [nigli@tamu.edu](mailto:nigli@tamu.edu). Students may receive feedback on sections of their MSE applications. Once the revisions are incorporated, the student will receive a formal letter from our Office notifying that their application was accepted.

**Table 1:** Medical Scholar Explorer Application Information

| **Student Name** |  | | | | | | **UIN** |  | | | | **Class Year** |  | | | |  |
| --- | --- | --- | --- | --- | --- | --- | --- | --- | --- | --- | --- | --- | --- | --- | --- | --- | --- |
| **Submission Date** |  | | | | | | **TAMU Campus** | | |  | | | | | | | |
| **Research Area** |  | | |  | |  | | | |  | |  |  | | |  | |
| **Affiliated Hospital** |  | | | | | | **Affiliated Site** | | |  | | | | | | | |
| **Address** |  | | | | | | | | | | | | | | | | |
| **Principal Investigator** | **Name** |  | |  | | | | |  | |  | | |  |  | | |
|  | **Job title** | |  | |  | | | |  | |  | | |  |  | | |
|  | **Institution** | |  | |  | | | |  | |  | | |  |  | | |
|  | **Department/Clinical specialty** | | | | | | | |  | |  | | |  |  | | |
|  | **E-mail** | | | | | | | |  | |  | | |  |  | | |
|  | **Website** | | |  | | | | |  | |  | | |  |  | | |

| 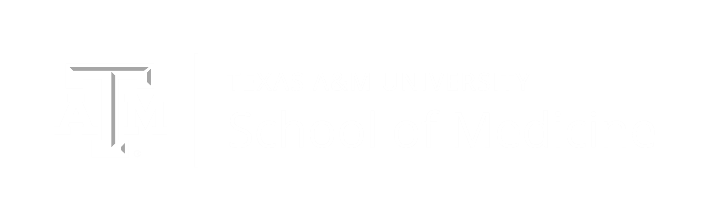 | MSRPP  *Medical Scholar Explorer (MSE)* |
| --- | --- |

**Table 2:** Medical Scholar Explorer Research Timeline

Students must specify whether their MSE pathway will follow a block (break, or approved School of Medicine leave of absence time) or a longitudinal timeline (1 semester or 2 semesters). Fill out the number of weeks planned for your **block MSE** in the table below. For a longitudinal MSE, provide an excel table as an *addendum* specifying the number of weeks you plan to spend completing your scholarly research project. If your plans change, you must notify as our Office as soon as possible, to update your research porfolio.

To strategically map your deliverables to your MSE project goals, students are strongly encouraged to discuss this timeline with your research mentor and come to an agreement on a defined research project. After you have been admitted to the MSRPP, you will be offered the choice to use *TimeClock plus* app to track your research hours. You are requested to submit your research hours to our Office, ***1 week*** before your end date.

| **Block MSE** | **Projected**  **Research time**  (hours/week) | **Actual**  **Research time**  (hours/week) |
| --- | --- | --- |
| **Start Date**  [MM,DD,YYYY] |  |  |
| **End Date**  [MM,DD,YYYY] |  |  |
| **Week 1**  [# h / date] |  |  |
| **Week 2**  [# h / date] |  |  |
| **Week 3**  [# h / date] |  |  |
| **Week 4**  [# h / date] |  |  |
| **Week 5**  [# h / date] |  |  |
| **Week 6**  [# h / date] |  |  |
| **Week 7**  [# h / date] |  |  |
| **Week 8**  [# h / date] |  |  |

| 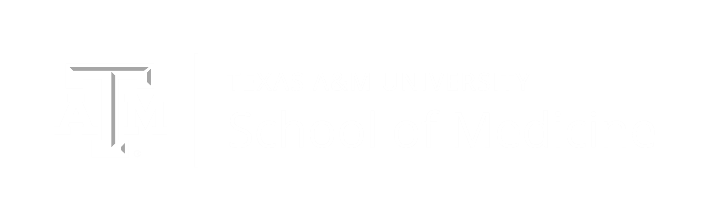 | MSRPP  *Medical Scholar Explorer (MSE)* |
| --- | --- |

**Table 3:** Medical Scholar Explorer Medical Educational Program Objectives

After meeting with the principal investigator, both student and mentor will collaborate to concretly delineate a MSE research project. Verify that the scope of your MSE research project clearly fits with the timeline specified in Table 2.

Students and mentors must write at least 1short-term and long-term goal/objective. Writing clear MEPOs is mandatory for your application to receive full consideration.

|  | **MSE Medical Education Program Objectives** |
| --- | --- |
| **Short-term Teaching Objective**  [To be filled by mentor] |  |
| **Long-term Teaching Objective**  [To be filled by mentor] |  |
| **Short-term Learning Objective**  [To be filled by student] |  |
| **Long-term Learning Objective**  [To be filled by student] |  |
| **Comments /**  **Questions** |  |

| 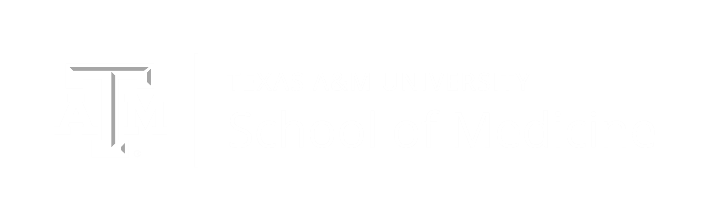 | MSRPP  *Medical Scholar Explorer (MSE)* |
| --- | --- |

**Table 4:** Medical Scholar Explorer Research Outcome and Deliverable Plans

Check the expected deliverable/outcomes for your MSE from the list below. You are strongly encouraged to give a final MSE *Launch Talk* research report to dissiminate your research to the entire School of Medicine community of scholars. Also, please specify the URL of the conference/workshop you plan to present your scholarly research project.

|  | **Outcome**  (check as appropriate) | | **Narrative**  (list title if applicable) | **Timeline**  (list estimated completion date) |
| --- | --- | --- | --- | --- |
| **Research skill 1**  [Short-term] |  | |  |  |
| **Research skill 2**  [Long-term] |  | |  |  |
| **Research report formal oral presentation**  [MSRPP launch talk series, and other invited talks] |  | |  |  |
| **Abstract/poster paper**  [Specify venue conference name and list URL, abstract deadline] |  | |  |  |
| **Discuss with your mentor what are you expected to contribue to the research project to gain co-authorship in a peer-reviewed publication**  [Specify targeted journal for your MSE research publication] | |  |  |  |
| **Will you write a capstone research report? I** |  | |  |  |

| 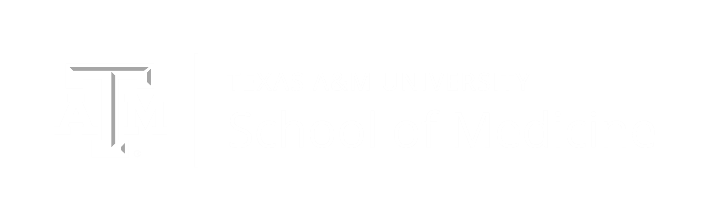 | MSRPP  *Medical Scholar Explorer (MSE)* |
| --- | --- |

**Medical Scholar Explorer Abstract**

Provide a synopsis of your planned MSE research project with a concise working hypothesis and a clear description of the unmet healthcare need that you would like to address. This narrative must include context details such a disease clinical symptoms, standard of care diagnosis criteria and treatment. Use font size 11 and technical medical language. Limit your abstract to 300 words.

| 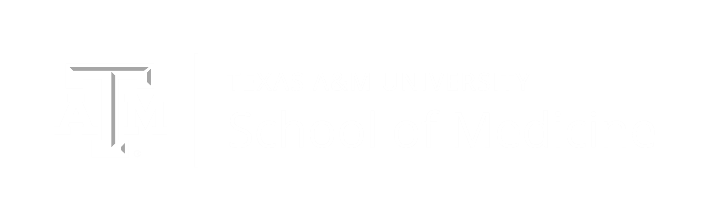 | MSRPP  *Medical Scholar Explorer (MSE)* |
| --- | --- |

**Medical Scholar Explorer Action plan Timeline Scheme**

Provide a flow-chart or visual aid for your weekly/montly action plan for your MSE research.

| 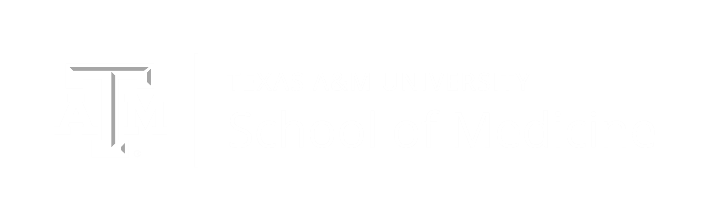 | MSRPP  *Medical Scholar Explorer (MSE)* |
| --- | --- |

**Medical Scholar Explorer Registration Signatures**

To complete your MSE application, collect the required signatures below. You must provide either a Department Head signature or a Clerkship Director signature. If neither is available, you may provide a Campus Dean signature (*). An e-mail from the PI or Department Head/Dean verifying knowledge of the student’s MSE participation are acceptable in lieu of paper /eletronic signatures sent to med-omsre@tamu.edu.

|  | **Printed Name** | **Signature** |
| --- | --- | --- |
| **Medical Student** |  |  |
| **Principal Investigator (PI)** |  |  |
| **Research Mentor**  (if different from PI) |  |  |
| **Department Head** |  |  |
| **Clerkship Director**  (if clerkship student) |  |  |
|  |  |  |
| **Campus Dean*** |  |  |

The Medical Student Research Education Office will document the completed MSE form in the research dossier of each Texas A&M School of Medicine medical student. Be pro-active and update our Office of any changes to your MSE research plan.

**Medical Scholar Explorer Assessment Survey**

Upon completion of your MSE, a short assessment survey will be sent to both the medical student and the principal investigator. Follow-up meetings of students and/or research mentor with Dr. Conover, the Director of Medical Student Research Education will evaluate whether the scope of scholarly research educational experience was met.

**Supplementary Fig. 1. Medical Scholar Explorer Application Form.** This is the form that was developed for medical students matched with faculty mentor to plan their MSRPP medical scholar explorer (MSE) research experience. Similar forms were developed for the Medical Scholar Researcher (MSR) and Distinguished Medical Scholar Researcher (DMSR) experiences with slight variations. For instance, MSR application it included a list of the research electives students registered for credit and for the DMSR application, it asked for the signature of the externship research director and the email acceptance for the externship program to fulfill the dedicated research year.

**Supplemental Fig. 2.** **Medical Student** **Personalized Coaching Research Sessions**. The number of contact hours and meeting of MSRPP candidates and admitted student were tracked during a 2-year period. Light grey areas marked with brackets show a ~ 25% increase in time and meetings to coach and teach individual MSRPP students in Spring 2022 as compared to any of the other 3 semesters. Meetings with the Director include in-depth discussion of a student motivation to pursue research, setting research goals with a timetable that fits academic schedule and future to further enhance students reasoning and critical thinking plus writing and oral presentation skills. A total of 645 contact coaching hours were tracked in individual 1:1 meeting with the Program Director in 381 one-on-one research coaching meetings during FY2020-2021 and FY2021-2022.

**
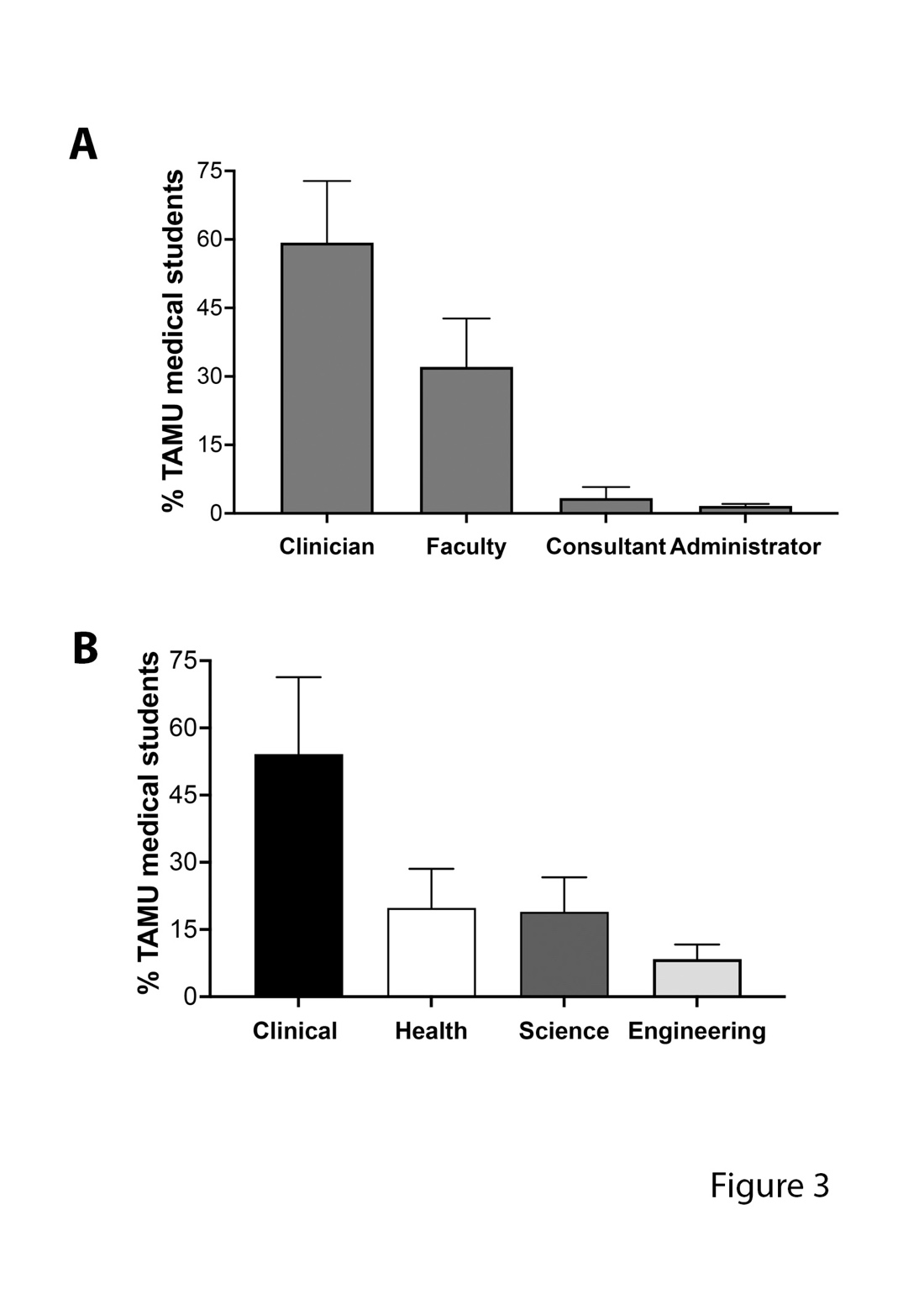
**

**Supplementary Fig. 3.** Perceptions of research motivation and fit with TAMU medical students. A research interest survey was administered to the entire medical student body (MS1 to MS4) to establish a baseline and get a sense of how research fits in their professional identity prior and during part of the implementation phase of the MSRPP program. This survey asked the entire student body about their research interests and motivations to participate in extracurricular research. Collective responses were analyzed from 4 separate anonymous surveys administered every 6 months during a 2-year period (September 2020 to August 2022). (A) In our learning environment, most students after graduation from medical students aspire to become clinicians, followed by a group that wants to incorporate scholarship and research, identifying as academic medicine faculty. (B) Most students want to be primarily involved in clinical research, as compared to those wanting to pursue research in other fields.


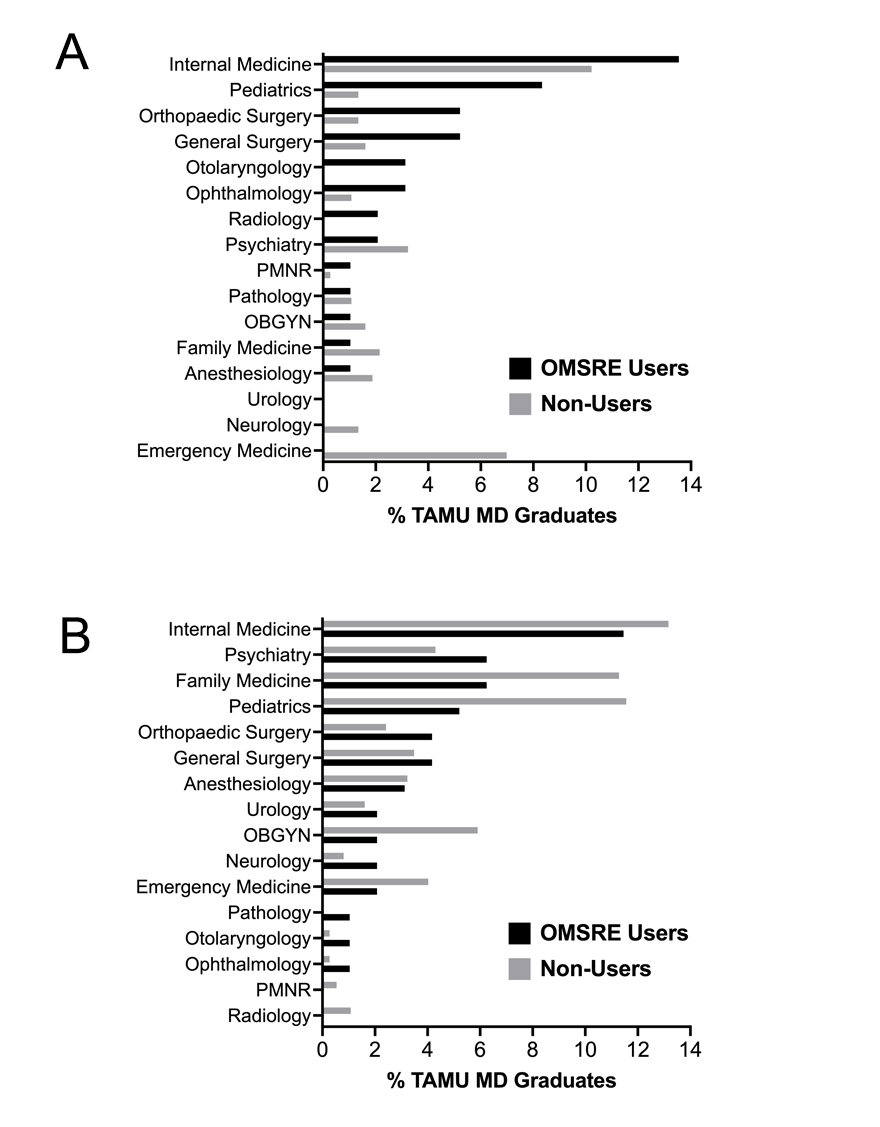


**Supplementary Fig. 4.** Impact of UME research training on residency match rates. Graphs show the percentages for TAMU MD graduates that used or not the services provided by the office of medical research education (OMSRE) research training programs sorted by specialty and location. This analysis excluded MD-PhD graduates and those who matched to preliminary and transitional year programs. During the study period (2020 through 2023), 47.9% of trainees matched to national residency programs while 52.1% matched to state residency programs. Black bars represent percentages of trainees that used OMSRE programs while grey bars show non-user in its initial 3 years of operation. (A) Bar graph displays the percentages of trainees that matched to national GME residencies. (B) Bar graph displays the percentages sorted by specialty of trainees that matched to state of Texas GME residencies.
